# Fucoxanthin Loaded in Palm Stearin- and Cholesterol-Based Solid Lipid Nanoparticle-Microcapsules, with Improved Stability and Bioavailability In Vivo

**DOI:** 10.3390/md20040237

**Published:** 2022-03-29

**Authors:** Yaxin Chen, Niaoniao He, Ting Yang, Shuyun Cai, Yi Zhang, Jinjing Lin, Mingqing Huang, Weizhu Chen, Yiping Zhang, Zhuan Hong

**Affiliations:** 1 College of Food Science, Fujian Agriculture and Forestry University, Fuzhou 350002, China; chenyaxin462@163.com (Y.C.); 13887434960@163.com (N.H.); zyifst@163.com (Y.Z.); 2 Technical Innovation Center for Utilization of Marine Biological Resources, Third Institute of Oceanography, Ministry of Natural Resources, Xiamen 361005, China; 18965156193@189.cn (T.Y.); sycai97@163.com (S.C.); 3 College of Pharmacy, Fujian University of Traditional Chinese Medicine, Fuzhou 350122, China; 2006027@fjtcm.edu.cn (J.L.); hmq1115@126.com (M.H.)

**Keywords:** fucoxanthin, microcapsule, solid lipid, antisolvent precipitation, stability, pharmacokinetic, bioavailability

## Abstract

Fucoxanthin (FX) is a marine carotenoid that has proven to be a promising marine drug due to the multiple bioactivities it possesses. However, the instability and poor bioavailability of FX greatly limit its application in pharmaceuticals or functional foods. In this study, the creative construction of a solid lipid nanoparticle-microcapsule delivery system using mixed lipids of palm stearin and cholesterol wrapped with gelatin/Arabic gum to load lipophilic FX was fabricated, aiming to improve the stability and bioavailability of FX. The results showed that the encapsulated efficiency (EE) and drug loading capacity (LC) of optimized FX microcapsules (FX-MCs) obtained were as high as 96.24 ± 4.60% and 0.85 ± 0.04%, respectively, after single-factor experiments. The average particle size was 1154 ± 54 nm with negative Zeta potential (−20.71 ± 0.93 mV) as depicted with size-zeta potential spectrometer. The differential scanning calorimeter (DSC) and thermogravimetric analyzer (TG) results indicated that FX-MC has a higher Tg and slower weight loss than FX monomers (FX crystal) and blank MCs. Besides, The Fourier transform infrared spectrometer (FTIR) confirmed the good double encapsulation of FX into the solid lipid and composite coacervate. Moreover, the encapsulated FX showed higher storage stability, sustained release (55.02 ± 2.80% release in 8 h), and significantly improved bioavailability (712.33%) when compared to free FX. The research results can provide a principle theoretical basis for the development and application of FX in pharmaceuticals or functional foods.

## 1. Introduction

Fucoxanthin (FX) is abundantly present in edible brown seaweed, and it is the second-largest marine carotenoid after astaxanthin [1]. Its unique propenyl group and some oxygen-containing functional groups enable it to have strong biological activity [2], including weight loss, lipid-lowering, anti-oxidation, anticancer, antihypertensive, and anti-inflammatory effects [3,4,5,6]. Thus, it has become one of the hotspots of algae functional resources research nowadays. However, FX exhibits poor stability [7,8,9], and it easily decomposes when exposed to light, heat, and oxygen under normal conditions [10]. In addition, FX cannot tolerate the low pH environment of human gastric fluid, and due to its lipophilicity, it is not easily absorbed in the water-based environment of the intestinal tract, leading to low bioavailability [11]. Pharmacokinetic studies have shown that when FX was administered to rats orally (65 mg/kg), its oral bioavailability was very low (0.06%) [12]. Besides, clinical trials have confirmed that after a certain dose of FX (0.52 mg/kg) was taken orally, the FX concentration in human plasma decreased (44.2 ng/L), which was only 33–46% in mice [13]. These existing problems greatly limit the application of FX in the production of functional foods.

Micro-nano delivery systems can improve the water solubility of FX, slow down the degradation caused by environmental factors, and achieve the purpose of protecting and improving bioavailability. Solid lipid nanoparticles (SLN) are one of the lipid-based delivery systems and a new type of microparticle encapsulation system developed in recent years [14,15]. SLN is a colloidal system composed of lipids (steroids, fatty acids, partial glycerides, waxes, and triglycerides), which are solid at room temperature [14]. It has the advantages of high stability, slow-release, and low toxicity [15], hence, is a promising novel drug delivery system carrier. A notable detail is that FX mixed with lipids could remarkably improve its bioavailability [16,17]. The mechanism is that since FX itself is lipophilic when it interacts with other oils, it could enhance the transportability through the cell membrane barrier and ultimately improve its bioavailability [18,19].

At present, most of the existing studies used a single solid lipid as the embedding carrier. For example, Quan et al. [19]. assembled FX-SLN with cetyl palmitate, and Li et al. [20] invented the use of stearic acid as the core lipid carrier of FX; although the fucoxanthin can be well protected, the undesirable encapsulated efficiency and poor drug loading capacity (LC) of its preparations are still the main reasons for limiting its large-scale production. Moreover, the solubility of FX crystals in oil is extremely low [21]. The principle is that it has strong solvent–solvent interactions, so that it is difficult to fall off from the compact crystal structure [22] and be successfully micellized. Additionally, the particle size of crystals is usually tens of micrometers, resulting in a greatly limited LC of the SLN. Complementary, the dissolution of FX crystals in lipids is usually accelerated by a high-temperature melting method [18], which is one of the current SLN preparation processes that not only easily degrades heat-sensitive FX, but around 40–50% cis-isomers would be generated [23,24], which is not conducive to absorption and utilization in the body, and greatly undermines the bioavailability of FX.

To overcome the above-mentioned deficiencies of the current technology, a simple but innovative strategy is to use mixed lipids—palm stearin (Ps) and cholesterol (Chol) as the FX lipid carrier—aiming to destroy the highly ordered crystalline structure of a single solid lipid and improve the drug-carrying capacity [20]. Ps is a by-product obtained after low-temperature crystallization of palm oil. In the preparation of MCs, Ps is considered to be the ideal core of solid lipid because of its high melting point, resistance to oxidation, and no trans-fatty acids [18]. One of the commonly used wall materials for preparing liposomes, Chol can adjust the fluidity of the phospholipid bilayer membrane [25], maintain the flexibility of the wall material, and reduce drug leakage; The anti-solvent precipitation [24]-binding ultrasound method can treat FX crystals at low temperature; under the premise of protecting the FX, a smaller particle size of FX microcrystal solution can be formed, which greatly promotes the dissolution of the FX in oil, effectively enhancing the LC [18], in the meanwhile significantly improving the bioavailability of FX in vivo.

To the best of our knowledge, the pharmacokinetics investigation on the relative in vivo bioavailability of FX-loaded MCs and FX crystal is still scarce. Moreover, the release properties and stability of FX-loaded MCs are worth investigating. To further enhance the water dispersibility and stability of FX-SLN, in this work, we creatively proposed to use the combination of lipid micro-nanoparticle technology and polymer MCs technology to fabricate an interesting solid lipid MC structure by the ultrasound-assisted anti-solvent precipitation method, that is, an FX-SLN core-wrapped gelatin/gum Arabic (Gel-GA) composite adhesive layer. In the next step, we focused on the impact of different process conditions on the properties of MCs, aiming to obtain FX-MC with controlled release, high stability, and high bioavailability. This study may therefore provide a theoretical basis for product research and development application of FX in the field of marine drugs or nutraceuticals.

## 2. Results and Discussion

### 2.1. Optimization of FX MC Preparation Process

#### 2.1.1. Effect of the Ratio of FX to Lipid and the Ratio of Core to Wall on FX MCs

**Figure 1:**
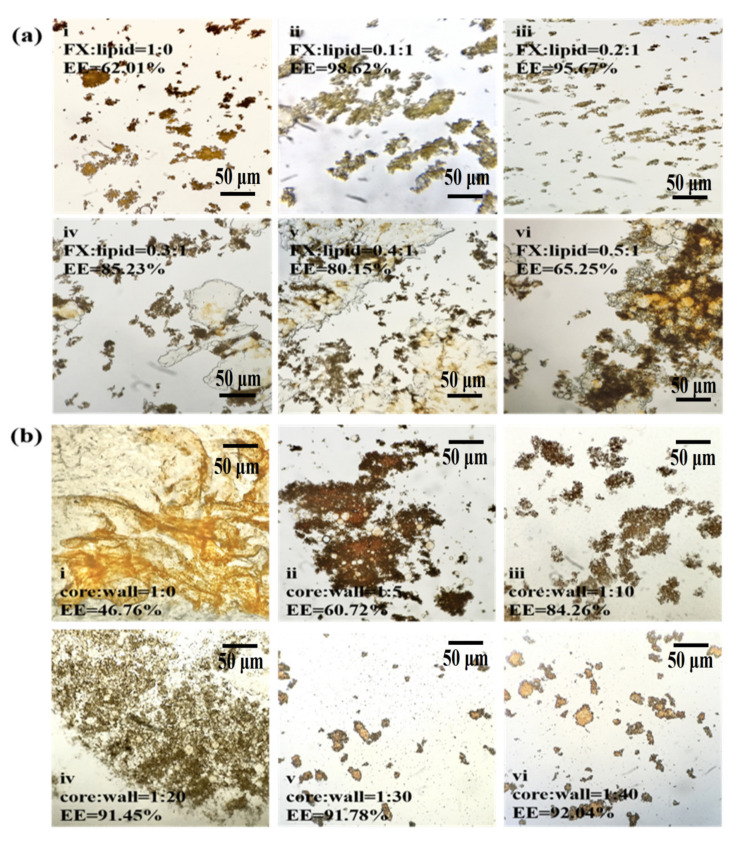
The effect of FX-to-lipid and the ratio of core-to-wall on EE of MCs. (**a**,**b**) The optical microscope images at different ratios of FX-to-lipid: (i) 1:0 *w/w*, (ii) 0.1:1 *w/w*, (iii) 0.2:1 *w/w*, (iv) 0.3:1 *w/w*, (v) 0.4:1 *w/w*, (vi) 0.5:1 *w/w* and different ratios of core-to-wall: (i) 1:0 *w/w*, (ii) 1:5 *w/w*, (iii) 1:10 *w/w*, (iv) 1:20 *w/w*, (v) 1:30 *w/w*, (vi) 1:40 *w/w*, and FX-GA-Gel (FX:Lipid = 1:0), FX-Ps-Chol (core:wall = 1:0) (at 100 or 400× magnification). EE represents the encapsulation efficiency of FX-loaded MCs.

The FX particles were reddish without lipid coating (Figure 1a(i)). Although the whole FX was in the form of agglomerated particles under the encapsulation of Gel/AG transparent gel film, the EE was only 62.01%. Immediately when the same lipid was added, as the amount of FX increased, the EE showed a downward trend. When the ratio of FX to lipid was greater than 0.3, free FX obviously existed in the gaps of the MC particles (Figure 1a(iv–vi)). The reason was that, due to the lipophilicity of FX, and under adequate emulsification, FX was evenly dispersed into the oil phase; as FX gradually increased, lipids of the same quality could not encapsulate all of it, and free FX increased [26]. Therefore, the EE of MCs was reduced.

Figure 1b shows that under the same core material quality, with the increase in wall material addition, the EE showed an upward trend (Figure 1b(i–vi)). At a core-to-wall ratio of 1:20 (Figure 1b(iv)), the EE was higher than 90%, which was significant (*p* < 0.01) when compared with the 1:5 core-to-wall ratio. Subsequently, the index stabilized (Figure 1b(iv–vii)), indicating that the content of the wall material wrapped in the core material became saturated. Therefore, 1:20–40 was selected as the appropriate range of FX-to-lipid ratios.

**Figure 2:**
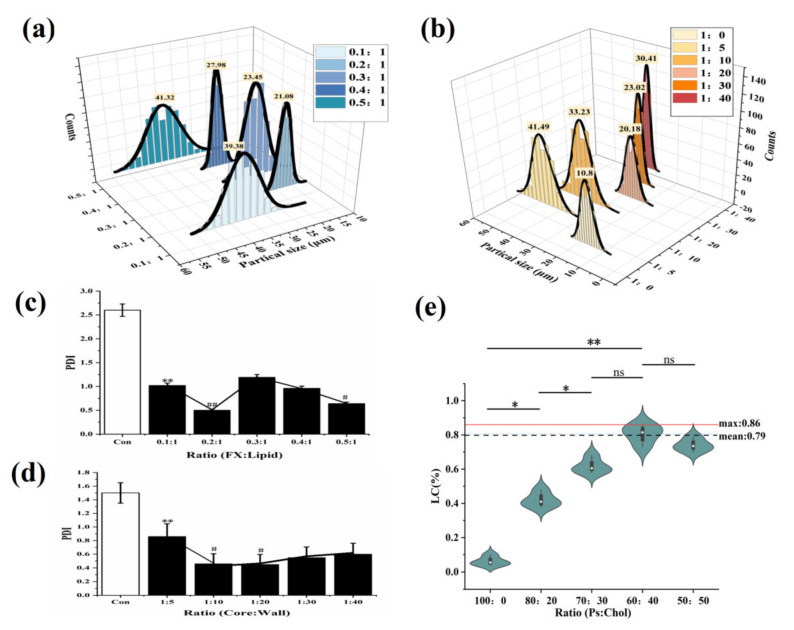
The effect of FX-to-lipid ratio and the ratio of core-to-wall on PDI and the impact of Ps to Chol ratios on the LC of MCs. (**a**,**b**) The hydration particle size of MCs with different FX-to-lipid ratios and core-to-wall ratios. (**c**,**d**) PDI of MCs with different FX-to-lipid ratios and core-to-wall ratios. * *p <* 0.05, ** *p <* 0.01 versus Con; ## *p <* 0.01, # *p <* 0.05 versus FX MC suspension at 0.1:1 or 1:5. (**e**) The drug LC of MCs with different ratios of Ps-to-Chol (The “ns” means there was no significant difference between groups).

In Figure 2a, under different FX-to-lipid ratios, the particle size first decreased and then gradually increased. When FX: lipid was 0.2:1 (*w/w*), the particle size was the smallest at 21.08 μm. While the FX was excessive, the interfacial tension of the solid lipid particle suspension increased, leading to agglomeration. In Figure 2b, the FX-SLN that formed without the addition of Gel-GA wall material had the smallest hydrated particle size of 10.8 μm. The addition of Gel-AG wall material could increase the particle size significantly (*p <* 0.05) and then decrease it. At the core-to-wall ratio of 1:20 (*w/w*), the MCs with the smallest particle size were obtained. Gel and GA possibly fully compounded and coagulated around the lipid to prevent the SLN from being exposed and adhered to each other, and this phenomenon was beneficial to reduce the viscosity of the system [26]. As the wall material continued to increase, the thickness of the particles increased, resulting in a larger particle size.

At the same time, the relationships between PDI and different FX-to-lipid ratios and different core wall ratios were investigated (Figure 2c,d). At a FX-to-lipid ratio of 0.2:1 (*w/w*) and a core-to-wall ratio of 1:20 (*w/w*), the MC suspensions with the lowest PDI value were prepared, indicating that the system with these conditions was the most stable, which was consistent with the particle size results obtained in Figure 2a,b. Therefore, we chose 0.2:1 (*w/w*) of FX: lipid as the best FX-to-lipid ratio, and 1:20 (*w/w*) of core: wall as the best core-to-wall ratio.

From Figure 2e, we observed that as cholesterol increased, LC then raised significantly (*p <* 0.01) until Ps:Chol was 60:40, and reached a maximum at that point, which was 0.79, nearly 20 times that of the case without Chol (LC was only 0.04%). This result confirmed that when compared with single Ps, the mixed solid lipid of Ps and Chol could achieve the purpose of obviously improving the LC of FX-MC.

#### 2.1.2. Effect of Aggregation pH and Ultrasonic Conditions on FX MCs

**Figure 3:**
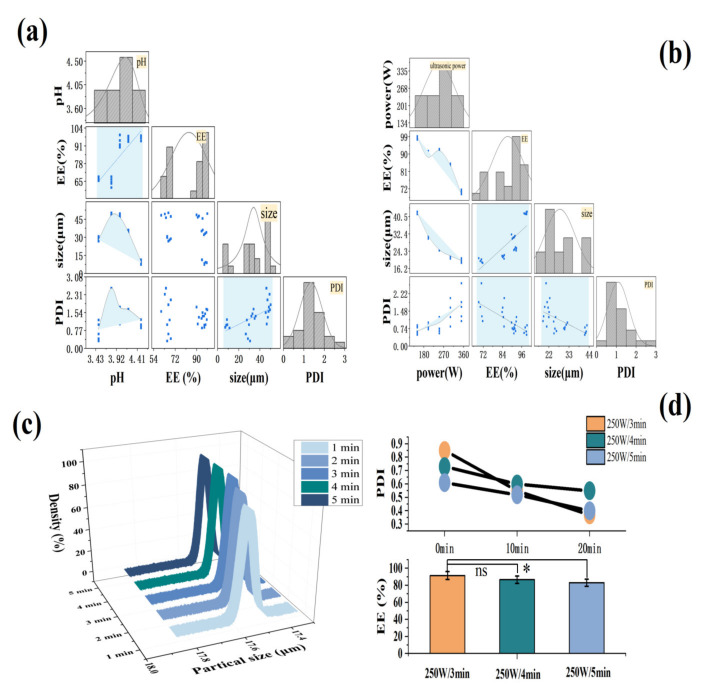
The effect of aggregation pH and ultrasonic conditions on MCs. (**a**,**b**) The relationship between MC’s EE, hydrated particle size, and PDI under different aggregation pH and ultrasonic powers. (**c**) The particle size distribution of the MCs under different ultrasound time. (**d**) MC PDI and EE under different ultrasound time. * *p <* 0.05; ns: no significant.

In Figure 3a, the EE was positively correlated with pH. At pH values greater than 3.8, EE was higher at greater than 90%, and the maximum EE was 96.48%. The hydrated particle size of the MCs also showed a good correlation with the pH value. The size first increased and then quickly decreased. The repelling charge in the system was speculated to be increased [27], causing the agglomerated particles to decrease with the increase in pH [28,29]. Meanwhile, more individual particles were dispersed in the suspension, and the result of a gradual decline in particle size was obtained, from 45.76 μm at pH 3.8 to approximately 11 μm at pH 4.5. The trend of PDI was similar to that of particle size, confirming that the number of individual particles increased, thereby improving the dispersibility of the MCs. In summary, pH 4.5 was selected as the optimal aggregation pH value.

As shown in Figure 3b, the EE and the ultrasonic power proved to have a good correlation, demonstrating an overall downward trend. At 150–250 W of ultrasonic power, the EE remained above 90%. The hydrated particle size and PDI of the MCs also were revealed to have a good correlation with the ultrasonic power. The particle size of the MCs became remarkably smaller with the increase in ultrasonic power, reducing from 40.76 μm to approximately 20 μm. At the same time, PDI increased, hence the smaller particle size made it easier for particles to contact each other [30], or the agglomeration occurred due to the inter-particle forces, such as van der Waals forces, which destroyed the stability of the system. When the ultrasonic power was in the range of 150–250 W, PDI < 1, which was judged as a relatively stable polymer dispersion coefficient [31]. At 250 W of ultrasonic power, the EE was relatively high (93.6%), and the particle size (18.6 μm) and PDI (0.85) reached the expected small value. Therefore, 250 W was selected as the best ultrasonic power. In addition, to obtain the best ultrasound time, the effect of different ultrasound times on the hydration particle size of the MCs was also investigated (Figure 3c). When the ultrasonic time was added to 3 min, the particle size of the MCs was reduced to 17.5 μm, and the subsequent increasing of ultrasonic power had no significant effect on the particle size (*p* > 0.05).

Figure 3d shows that the PDI value of the MCs decreased the most with the increase in stirring time under the power of 250 W for 3 min, followed by ultrasound for 5 min, and ultrasound for 4 min had the least loss. The EE was in line with this. Consequently, the ultrasonic power of 250 W and the time of 3 min were considered as the best ultrasonic conditions, where the result of EE and LC were 96.24% and 0.85% (data not shown), respectively. The high EE suggested that most of FX had been adequately protected, and the LC of optimized MC in our work, compared to the LC of 0.04% obtained in the previous study by Wang et al. [18], had been obviously improved. This may be because of mixed Ps/Chol, as the solid lipid core, as well as the optimizing preparation conditions, lead to significantly high LC of FX MC.

### 2.2. Characterization of FX MCs

#### 2.2.1. Surface Morphology, Particle Size, and Zeta Potential Analysis

Whether FX MCs were formed was determined by observing the morphological difference of FX before and after embedding via SEM.

**Figure 4:**
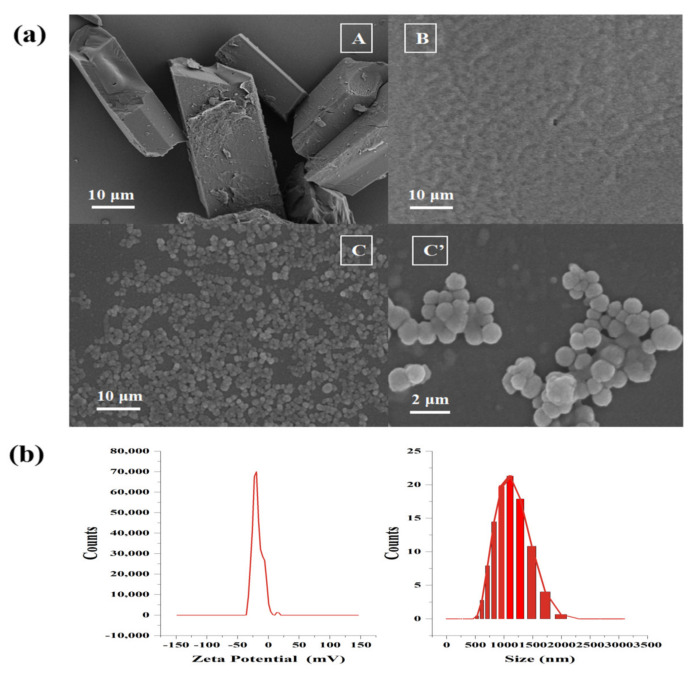
SEM (**a**) of FX crystal (**A**), blank MCs (**B**), and FX MCs (**C**), (**C**′) at different magnifications. The particle size and Zeta potential of the FX MCs with pH = 7.0 (**b**).

In Figure 4a(A), due to the low water solubility of FX, the FX crystal remained an inherent cuboid shape in the water, and the overall average length was more than 50 μm. The unloaded MCs (Figure 4a(B)) were gel particles formed by the complex cohesion of Gel and GA, with a particle size less than 500 nm. When the blank MCs were dissolved in water and dried for observation, in consideration of the water solubility of the wall material, the gel particles were easy to combine with each other, to form irregular aggregates. In Figure 4a(C), the FX MCs showed spherical shapes. Under 10 K magnification, the surface was relatively smooth (Figure 4a(C′)) with no cracks, hinting that the surface of FX-SLN had been wrapped completely by the wall material through compound condensation. In addition, the tannins could also enhance the stability of MCs, preventing GEL/GA from decomposing. 

As observed in these micrographs, the FX crystals, blank MCs, and FX MCs showed different forms, suggesting that the FX-loaded MC was a new substance, indicating that the MC loaded with FX has been formed [31].

In addition, the FX MCs’ particle size distribution was a narrow curve with a mean particle size of 1154 nm, and with a negative surface charge (around −20.71 mv), as shown in Figure 4b, suggesting that the size of FX MCs met the micron level and the suspension of FX MC had good stability [32,33]. 

#### 2.2.2. DSC Analysis of Materials

**Figure 5:**
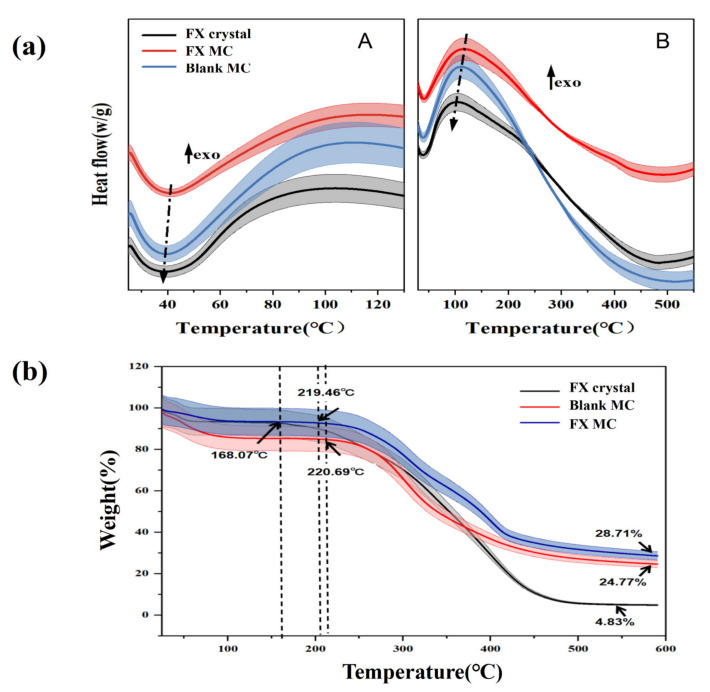
(**a**) DSC thermograms of FX crystal, FX MC, and blank MC. (The arrow with “exo” means the exothermic direction; The dotted line indicates the direction of temperature change). (**b**) TG curve of FX crystal, FX MC, and blank MC. (The broad colored curved shadow means the range of error.).

The thermodynamic properties of FX crystal, blank MC, and FX-MC were compared by DSC, as shown in Figure 5a. Tg is exhibited by amorphous regions of the crystalline polymer change from the glassy state to the viscous rubbery phase or the latter transitions to the former [34]. Generally, the higher the Tg transition temperature, the better the temperature resistance of the product [35]. Therefore, when developing new products based on polymers, this characteristic should be highly considered. 

In Figure A, we could see that the Tg of the three substances all moved to the left. Among them, the Tg of FX crystal was less than 40 °C (about 38 °C), which is close to body temperature, so it was easy to produce the fragile crystal structure in the human body, and its mechanical strength was weakened, which was likely to be degraded. In contrast, the Tg of FX-MC was higher than 40 °C, which indicated that this polymer can show a rather hard chain structure and improve the stability of FX. One underlying reason was that negatively charged Gel and positively charged GA formed a gel network structure through complex cohesion to load FX [36]. Another reason was the crystallization characteristics of solid lipid, which made the Ps/Chol lipid core form a crystal structure with a certain strength [37]. Although the Tg of blank MCs was slightly lower than 40 °C, it was still higher than that of the FX crystal, which suggested that the SLN-MC carrier had a certain thermal protection effect on FX. 

In Figure B, it was observed that the overall peak temperatures of the three substances moved to the left, and the enthalpy of FX-MC was the highest, which manifested in the phase transition (from the highly elastic phase to the glass phase) requiring the highest heat. This result was identical to Figure 5a, thus, FX-MC had better thermal stability.

#### 2.2.3. Thermogravimetric (TG) Analysis of Materials

Figure 5b shows the thermal curve obtained from the TG analysis of FX, blank MC, and FX MC. The thermal behavior was studied at 40–580 °C.

From 40 to 100 °C, slight weight loss was found in all three samples, possibly due to the desorption of captured CO_2_, evaporation of residual solvent, or related to the initial decomposition of the core material and wall material [38], similar to the results in Figure 5a(A) of DSC.

A significant weight loss zone (45.06%) was observed from 150 to 266 °C, which was attributed to the decomposition and evaporation of Gel and GA polymers. For FX crystals, the initial evaporation temperature was 168.07 °C, which was lower than that of blank MCs (220.69 °C), and the initial degradation temperature of FX-loaded MCs was similar to that of blank MCs (219.46 °C). Their initial degradation temperature was significantly higher than that of the FX crystal, indicating that FX MCs could improve the thermal stability of FX crystal.

At approximately 550 °C, the three all reached the maximum degradation rate. At this time, the thermal weight loss of FX crystal remained constant, indicating that the end of thermal decomposition, and the weight loss was as high as 95.17%. Moreover, it was observed that the weight loss of blank MCs (75.23%) and FX MCs (71.29%) were close, which was speculated that because there was no strong chemical interaction, which could change the chemical structure and structural integrity of the FX between the wall material and FX core material. Therefore, there might be a physical combination between FX mixed lipid core and polymer wall material.

#### 2.2.4. FTIR Analysis of Materials

FTIR is used to determine the degradation of the polymer matrix in the carrier system [39]. In addition, FTIR can be used to characterize the chemical structure of materials and the interaction between polymers and drugs [40]. 

**Figure 6:**
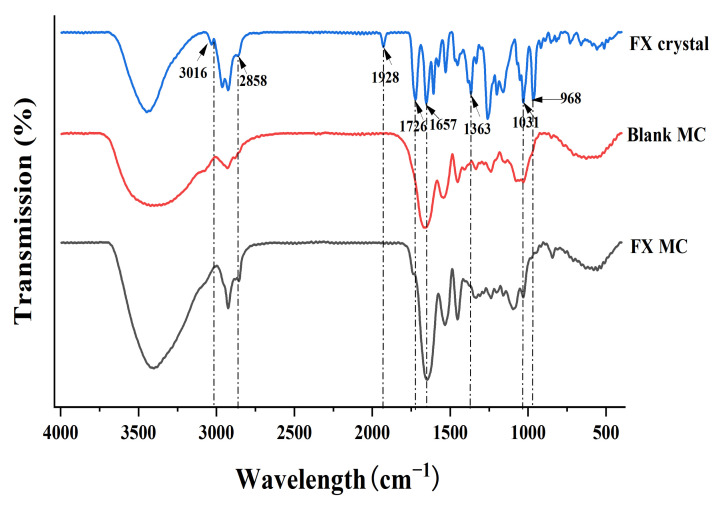
FTIR spectra of FX crystal, blank MC, and FX MC.

In this study, FX crystal, blank MC, and FX MC were evaluated by the FTIR method, and the obtained spectrum is shown in Figure 6.

For a pure FX sample (FX crystal), the infrared absorption spectrum had a characteristic peak at 1928 cm^−1^, which was the characteristic peak contributed by FX’s unique functional group allene bond (C=C=C). Beyond that, the peak at 2858 cm^−1^ referred to the stretching vibration of -CH, the peak at 1363 cm^−1^ represented the bending vibration of -CH, and the peak at 3016 cm^−1^ signified the bending vibration of =CH, which were all characteristic peaks of carotenoids [34].

The peak values of 1726 (-C=O stretch) and 1031 (-C-O stretch) cm^−1^ were the characteristic peak of esters, while 1657 cm^−1^ indicated the presence of ketones (C=O). In addition, the peak value of 968 cm^−1^ was the absorption peak of the out-of-plane bending vibrations from the C-H bond in the C-C conjugated system, which was the characteristic peak of trans-substituted ethylene. However, in the sample of FX-MCs, the characteristic peaks of FX at 1928, 1726, 1657, and 968 cm^−1^ did not appear, which showed that FX had been successfully embedded in the SLN-MC carrier. There was also no new absorption peak was observed, which indicated that FX was loaded into the wall material shell by physical intercalation rather than chemical cross-linking, which was basically consistent with the previous TG analysis results, so the chemical properties, especially the biological activity of FX, have not been changed.

### 2.3. Performance Analysis of FX MCs

#### 2.3.1. In Vitro Release Study

**Figure 7:**
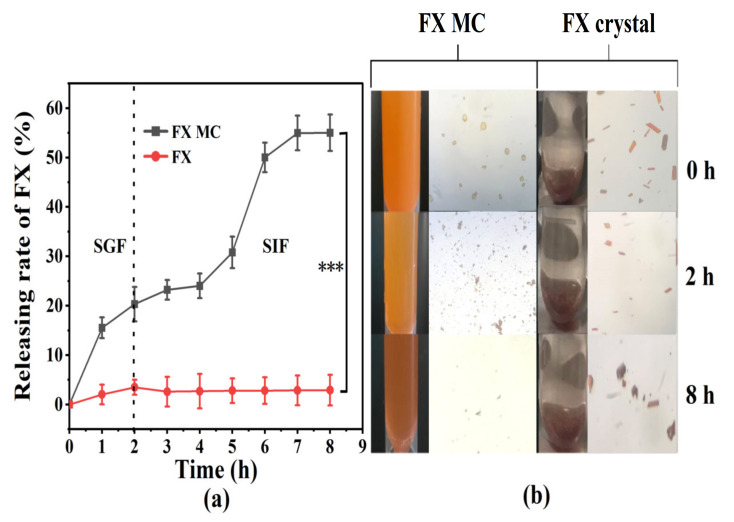
(**a**) In vitro release curve of FX MCs and FX crystals in simulated gastrointestinal fluid. (**b**) The optical microscope photos of the FX MCs and FX crystals digestive fluid taken at 0, 2 h (GIF digestion stage), and 8 h (SIF digestion stage), respectively. *** *p <* 0.001.

In the SGF stage, the FX was shown to be slowly released from MCs, and the cumulative amount of FX released was 20.33% (Figure 7a). No sudden release was observed from the overall release curve, which confirmed that FX was uniformly dispersed in the lipid mixture inside, instead of the MC’s surface. The MC was destroyed during the incubation of SGF, and then the encapsulated FX-SLN core was slowly released. That was because the low pH environment (pH = 2) of SGF could partially protonate the GA, thereby destroying the electrostatic adsorption between the shell materials, and pepsin could also hydrolyze wall materials. The yellow deepening of digestive fluid was observed visually, which was because the yellowish SLN core was completely exposed after the shell material dissolved and collapsed in a water-based environment. Meanwhile, the optical microscope images (Figure 7b) clearly showed that the shape of the MCs degraded from the original nearly circular shape to irregular flocculent particles.

In the SIF environment, the release of FX from FX MC significantly increased (*p* < 0.001), reaching 55.02% (Figure 7a), and the color gradually became red (Figure 7b), which was attributed to the further digestion and dissociation of the solid lipid core, which released the red FX. Furthermore, MC particles could hardly be observed from the optical microscope image at 8 h (Figure 7b), which also confirmed that the MCs were almost completely degraded. 

When compared with FX MCs, only approximately 3.50% of FX crystals were released from the dialysis bag during the incubation in SGF (Figure 7a), and the color of the SGF was close to transparent (Figure 7b). The potential reason was supposed to be the water-based characteristic of SGF, making FX difficult to dissolve and release. The optical microscope image at 2 h (Figure 7b) demonstrated no obvious change in the color and size of the FX crystal when compared with the original state, which confirmed the previous speculation as well. Besides, the optical microscope image of SIF at 8 h exhibited that the crystal size was visibly reduced, but after centrifugation, the FX in the supernatant was still almost undetectable. 

Based on the above experimental results, it was speculated that when FX MC was administered in vivo, the SLN-MC carrier could protect FX from passing through the stomach and release FX obviously in the small intestine, thereby enhancing the efficacy of FX.

#### 2.3.2. Accelerated Stability Study

**Figure 8:**
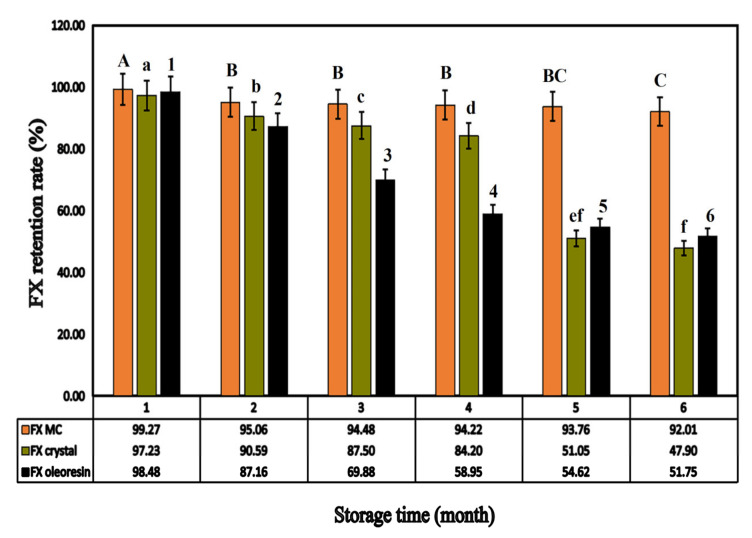
The FX retention of different FX preparations (FX MC, FX crystal, FX oleoresin) depending on the storage time. (Different letters indicate a significant difference, *p* < 0.05).

Under the same storage conditions, unembedded FX preparations (FX crystals and FX oleoresin) were selected as the control group for accelerated stability studies. As shown in Figure 8, during the storage process, the FX retention rates of the three formulations all showed a significant downward trend (*p* < 0.05). By month six, the content of FX in crystals was only 47.90%, which was a loss of more than half. Moreover, the FX in oleoresin degraded to 51.75%, which also showed a significant difference in FX content (*p* < 0.05). The results showed that the stability of the two formulations in the control group was poor.

In contrast, after the acceleration period (0–6 months), the retention rate of FX in FX MC was detected to be 92.01%, with a non-obvious decline, which was 8%, and it implied that FX was relatively stable, mainly due to the prevention of FX leakage by Ps/Chol solid lipid carrier and protection of GEL-GA coacervate shell structure through cross-linking [41], which could improve the stability of FX.

#### 2.3.3. In Vivo Bioavailability Studies of FX MCs Compared with FX Crystals

**Figure 9:**
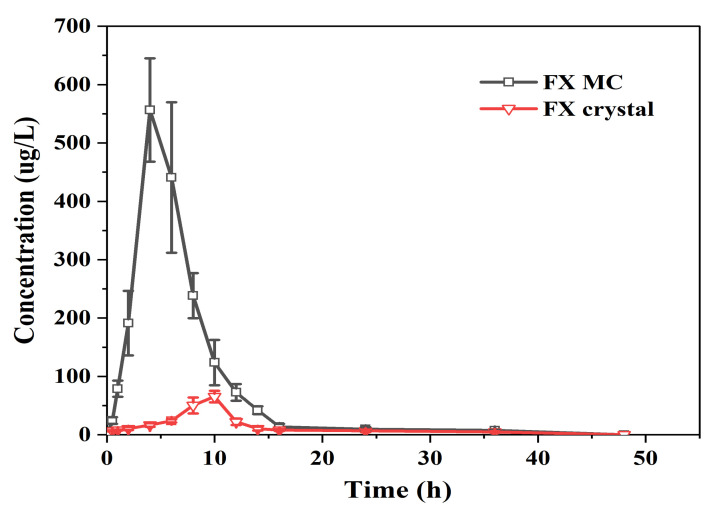
Mean drug concentration in plasma–time curves of FXOH in rats following administration of FX MCs and FX crystals (25 mg/kg FX/body weight) (*n* = 6; mean ± SD).

Studies on the digestion, absorption, and metabolism of dietary FX showed that FX was hydrolyzed in the gastrointestinal tract or the intestinal absorption process and deacetylated by the hydrolysis of lipase and Chol enzyme; then, FXOH was used as a primary metabolite to be incorporated into the blood [42]. Thus, the plasma was measured using FXOH to compare the bioavailability of different FX preparations. The absorption kinetic parameters of FXOH after a single gavage of FX crystals and FX MCs in rats are shown in Table 1, and the drug concentration in a plasma–time curve is shown in Figure 9.

When the dose of FX crystal reached 25 mg/kg, the drug concentration in plasma was very low, the T_max_ was 9.33 ± 0.81 h, and the C_max_ was only 65.42 ± 9.08 ng/mL. The FX MC reached the T_max_ at 4.67 ± 1.03 h, and the C_max_ attained 556.60 ± 46.20 ng/mL, which was eight to nine times the absorption of the raw material (FX crystal).

The fitting results of pharmacokinetic parameters showed that the T_1/2_ of the FX crystal and FX MC were 11.30 ± 2.29 and 7.80 ± 0.75 h, respectively, and the MRT_0–t_ were 12.87 ± 0.53 and 7.01 ± 0.41 h, respectively. The embedded FX shortened the T_1/2_ and MRT_0–t_ of the FX crystals, which may be because of the better solubilization effect of the FX MC preparation on FX, which increased the utilization rate, followed by a significant increase in C_max_. The AUC_0-48_ of FX crystal was 493.86 ± 40.12 (ng/mL) h. In contrast, the AUC_0-48_ of FX MC reached 3517.89 ± 272.53 (ng/mL)·h, which was markedly higher than that of FX crystals (*p* < 0.01). According to calculations, the oral relative bioavailability of FX MC was higher, at 712.33%, than that of FX crystals by approximately seven times, and the statistical analysis showed there was an obviously significant difference when compared to FX crystals (*p* < 0.01), indicating that the oral bioavailability of FX in vivo was greatly improved. Two reasons could be speculated. Firstly, the addition of lipids (Ps and Chol) made the FX easier to penetrate the biofilm [43]. Secondly, the anti-solvent precipitation combined with ultrasonic treatment can make FX form into an amorphous state that can easily form micelles with hydrophilic polymer wall material, which are able to be absorbed by intestinal epithelial cells [44].

## 3. Materials and Methods

### 3.1. Materials and Animals

The FX (FX crystal) and fucoxanthinol (FXOH) were prepared and identified by UV, MS, and NMR spectroscopies in the laboratory as described previously (purity ≥99%) [12]. For the preparation and characterization of MCs, Ps was purchased from Mengqi Technology Co., Ltd. (Wuhan, China). Chol, trypsin, and dialysis bags (500–1000 molecular weight cutoff) were purchased from Yuanye Biology Co., Ltd. (Hunan, China). Pepsin was obtained from Sinopharm Co., Ltd. (Shanghai, China). Tannin, Gel, and GA were purchased from Macklin Co., Ltd. (Shanghai, China), and they were all food-grade. HPLC-grade acetonitrile and methanol were obtained from Merck KGaA (Darmstadt, Germany). HPLC-grade formic acid was obtained from Roe Scientific Inc (Powell, OH, USA). Ultrapure water was produced by a Millipore Milli-Q system (Millipore Corp., Billerica, MA, USA). All the reagents or solvents were commercially available and of reagent grade. Healthy rats weighing 200 ± 20 g were purchased from the Laboratory Animal Centre of Xiamen University (Xiamen, China).

### 3.2. Preparation of FX MCs

FX MCs were prepared in accordance with Wang et al. [18], with some modifications. Chol and Ps (Chol: Ps = 0.6:1 *w/w*) were melted in acacia solution (1.5%, *w/v*) at 55 °C. After cooling to 38℃, 1 mL of ethanol containing FX crystals was slowly added to the solution (FX: mixed lipid = 0.2:1 *w/w*). By this time, the FX ethanol solution was precipitated in the water due to the effect of the antisolvent. Under an ice bath, ultrasonic crushing treatment was performed at a power of 250 W for 3 min, and the temperature was controlled as to not to exceed 38 °C during the process. FX crystals with small particle size could be obtained, and an FX solid lipid nanoparticle suspension was formed. The suspension was mixed with an equal volume of Gel solution (1.5%, *w/v*) and homogenized at a high speed of 20,000 rpm for 8 min. Complex coacervation reaction was performed to form FX-Ps/Chol-GA/GE MCs (FX-mixed lipid core: wall material = 1:25, *w/w*). Acetic acid (10%) was used to adjust the pH to 3.5–4.5, magnetically stirred under an ice bath at 500 rpm, and cooled to below 10 °C. Tannic acid solution (18%, total mass fraction of 0.4%) was slowly added onto the surface of the MCs to be cured. It was let to stand for 30 min, and after centrifugation (8000 rpm, 10 min), the precipitate was washed and freeze-dried to obtain FX MCs.

### 3.3. Optimization of the Preparation

The ratio of Ps and Chol on LC of FX MCs, the ratio of FX to mixed lipids (Ps and Chol), the ratio of core material to wall material, the pH value of aggregation, and the effect of ultrasonic conditions (including power and time) on the EE, particle size, and PDI of FX MCs were studied separately. The experimental methods were shown as follows:

#### 3.3.1. Effect of Ps-to-Chol Ratios on FX MCs

The ratios of Ps-to-Chol were 100:0, 80:20, 70:30, 60:40, and 50:50 (*w/w*). Other process conditions were carried out as described in Section 3.2.

#### 3.3.2. Effect of FX-to-Lipid Ratios on FX MCs

The ratios of FX-to-lipid were 0.01:1, 0.1:1, 0.2:1, 0.3:1, and 0.4:1 (*w/w*). Other process conditions were carried out as described in Section 3.2.

#### 3.3.3. Effect of Core-to-Wall Ratios on FX MCs

The ratios of core-to-wall were 1:5, 1:10, 1:20, 1:30, and 1:40 (*w/w*). Other process conditions were carried out as described in Section 3.2. 

#### 3.3.4. Effect of Aggregation pH on FX MCs

The pH value was adjusted to 3.5, 3.8, 4, 4.2, and 4.5. Other process conditions were carried out as described in Section 3.2.

#### 3.3.5. Effect of Ultrasound Conditions on FX MCs 

The ultrasonic powers were 150, 200, 250, 300, and 350 W, and the ultrasonic times were 5, 4, 3, 2, and 1 min. Other process conditions were carried out as described in Section 3.2.

Through single factor experiments, three indicators of EE, particle size, and PDI were comprehensively evaluated, and the optimized process conditions of FX MCs were selected as the best preparation methods.

### 3.4. HPLC Analysis of FX

After filtering was performed with a 0.22 μm membrane filter, the sample (10 μL) was injected into the HPLC system (Agilent 1260, Shimadzu, Japan) and passed through an Agilent TCC18 column (4.6 mm × 250 mm, 5 μm) to be detected by a UV/Vis detector. Methanol–water (92:8) was used as the mobile phase at a flow rate of 1 mL/min and a column temperature of 30 °C. The FX was detected at 450 nm [45].

### 3.5. Determination of EE

For the determination of free FX on the surface of MCs, 2 mg of wet FX MCs was accurately weighed and placed in a 10 mL graduated centrifuge tube. Then, 5 mL of petroleum ether (boiling range of 30–60 °C) was added with a vortex for 3 min. After centrifugation for 10 min at 8000 rpm, the supernatant was accurately diluted with anhydrous ethanol, and the FX peak was measured at 460 nm to calculate the amount of FX on the surface of the MCs.

For the determination of total FX in the MCs, 2 mg of wet FX MCs was accurately weighed, 5 mL phosphate buffer (pH 7.0) was added with 0.1 mg neutral protease to break the wall material of MCs, and the solution was then poured into a rotary evaporation flask and swirled until the liquid was evaporated. Afterward, the rotary evaporation flask was washed with 4 mL anhydrous ethanol. It was centrifuged (3 min, 8000 rpm) and the supernatant was diluted for testing. The EE of FX was evaluated as follows [46]:

(1) $$\mathrm{EE}(\%)=\left(1-\frac{\mathrm{free}\;\mathrm{FX}\;\mathrm{on}\;\mathrm{the}\;\mathrm{surface}\;\mathrm{of}\;\mathrm{MCs}}{\mathrm{total}\;\mathrm{FX}\;\mathrm{of}\;\mathrm{MCs}}\right)\times 100\%$$

### 3.6. Determination of Drug LC

The FX MCs were accurately weighed to determine the content of FX. The measurement method was the same as that for total FX determination in 3.5. LC was calculated using the following formula [46]:

(2) $$\mathrm{LC}(\%)=\frac{\mathrm{FX}\;\mathrm{content}\;\mathrm{in}\;\mathrm{MCs}}{\mathrm{total}\;\mathrm{weight}\;\mathrm{of}\;\mathrm{MCs}}\times 100\%$$

### 3.7. Characterization of FX MCs

#### 3.7.1. Surface Morphology Analysis

The surface morphology and microstructures of FX MCs, blank MCs, and FX crystals were imaged by SEM (JSM-6490LV, JEOL Ltd., Tokyo, Japen). The test conditions were 2.00 K × magnification, acceleration voltage 3 kV [26]. In order to obtain the microscopic morphology, the FX MCs were also observed at a magnification of 4.00 K ×.

#### 3.7.2. Particle Size, PDI, and Zeta Potential Analysis

The particle size, PDI, and zeta potential were measured by dynamic light scattering technology (DLS) using a Zeta-sizer Nano ZS90 (Malvern Instruments Ltd., Malvern, UK) at 25 °C and pH 7. The number of measurements was adjusted three times, taking the average value as the final data.

#### 3.7.3. Thermodynamic Properties Analysis

Tests were carried out on FX crystals, blank MCs, and FX MCs via DSC (Mettler Toledo 200 N, Mettler Inc., Zurich, Switzerland) to study the thermal stability of the samples. An amount of 4 mg of each sample was accurately weighed, spread evenly in the aluminum dish, sealed with a capping machine, and tested with an empty aluminum dish as a control. The measurement was performed under nitrogen as the purge gas from 20–240 °C at a heating rate of 10 °C/min [34].

TG analysis of the FX crystals, blank MCs, and FX MCs was performed on the Q5000IR thermogravimetric analyzer (PYRIS Diamond TGA, Perkin Elmer Inc., Waltham, MA, USA) to investigate the thermal behavior. The degradation rate was observed at high temperatures, from 25 to 800 °C, at a heating rate of 20 °C/min, under the carrier gas of high-purity nitrogen, during which the flow rate reached 20 mL/min [34].

#### 3.7.4. Functional Group Analysis

FTIR (Spectrum100, Perkin Elmer Inc., Waltham, MA, USA) was used to characterize the functional group analysis of the samples (FX crystals, blank MCs, and FX MCs). The samples were measured by KBr tableting method, that is, 100 mg of KBr and 1 mg of sample were completely ground, mixed in a dry state, filmed, and scanned. The test condition was that the scanning wavelength range was 400–4000 cm^−1^ under 4 cm^−1^ resolution. The combination of FX and wall materials was judged depending on the information about the composition of samples provided by FTIR [9].

### 3.8. Performance Evaluation of FX MCs 

#### 3.8.1. In Vitro Release Study

The formula of human gastric fluid and intestinal fluid was stimulated in accordance with Aditya et al. [47] For the stomach liquid preparation, pepsin (0.32% *w/v*), sodium chloride (2 g), and concentrated hydrochloric acid (7 mL) were dissolved in 1 L water, and the pH was adjusted to 2.0 ± 0.1 with 0.1 mol/L hydrochloric acid. For the intestinal fluid preparation, trypsin was dissolved in phosphate buffer to make the final concentration 0.5 mg/mL, and the pH was adjusted to 7.2 ± 0.1 with 1 mol/L sodium hydroxide. Both of them were ready to use.

An appropriate amount of ethanol (60% *v/v*, of digestive fluid) is necessary to be added to the gastric and intestinal fluids to improve the release of FX in a water-based environment. A dialysis bag containing 20 mg of FX MCs was placed in a beaker containing 25 mL of gastric fluid and then placed on a shaker at 37 °C and 120 r/min. The samples were taken at the specified time during this process. After 2 h, 25 mL of intestinal fluid was added to the Erlenmeyer flask, and the pH was adjusted to 7.2 ± 0.1 with 1 mol/L sodium hydroxide. Incubation was continued for 8 h, and the samples were taken at a fixed time, using unembedded FX (FX crystals) as a control. Depending upon the 3.4 HPLC method to detect the content of FX, the release rate of FX was determined as follows:

(3) $$\mathrm{FX}\;\mathrm{release}(\%)=\frac{M_{0}-M_{t}}{M_{0}}\times 100\%$$

where M_0_ is the amount of FX initially encapsulated, and M_t_ is the amount of FX remaining in the microspheres at a given incubation time, t.

#### 3.8.2. Accelerated Stability Study

The accelerated stability evaluation was based on the method mentioned by Ruben et al. [48] to evaluate the stability of the product. The specific content was as follows:

A 6 month stability investigation was conducted on the retention of FX in FX preparations. Accurately weighed (40 mg) MCs were placed in a 3 mL glass bottle, sealed, and then stored in the dark at a relative humidity of 65% under 25 °C. Samples were tested every month, and the mass of FX remaining in the MCs was determined as described in Section 3.5. The retention rate of FX in the MCs was obtained as follows:

(4) $$\mathrm{FX}\;\mathrm{retention}(\%)=\frac{M_{t}}{M_{0}}\times 100\%$$

where M_t_ denotes the FX content after storage for a period of time, and M_0_ refers to the original FX content.

Accurately weighed crystals and oleoresin with the same quality were placed into a glass bottle and stored under the same conditions. Both of them were used as controls.

#### 3.8.3. In Vivo Bioavailability Study

The in vivo bioavailability of FX MCs was determined via pharmacokinetic study in rats, as referenced from a previous study [21] but with some adaptive adjustments.

FX MCs and FX crystals were dissolved in 0.5% CMC solution and administered as a suspension. Twelve clean-grade male SD rats were selected and randomly divided into two groups. The rats fasted for more than 12 h before administration, and blank blood was taken. One group was given FX MC suspension, and the other group was given FX crystal suspension. The dosage of both was 25 mg/kg (based on FX). Then, 0.5, 1, 2, 4, 6, 8, 10, 12, 14, 16, 24, 36, and 48 h after administration of each group, blood was collected from the fundus vein and placed in a heparinized EP tube, centrifuged (4000 r/min) for 10 min, and stored in the refrigerator at −20℃ after separating the plasma sample for testing. Hereafter, 100 μL of plasma was accurately transferred into a 2 mL EP tube. Then, 740 μL of methanol was precisely added and vortexed for 3 min for subsequent extraction. Centrifugation was conducted at 12,000 rpm for 10 min, and the supernatant was filtered with a 0.22μm filter membrane for FX content analysis by LC-MS.

### 3.9. LC-MS Analysis of FX Metabolites

The determination of FX metabolites in rat plasma was in accordance with a study described previously [21]. Analyte separations were performed on an Agilent UPLC-1290 system (Agilent Corp., Milford, MA, USA) by using an SB-C18 column (2.1 × 100 mm, 1.8 m, Agilent Technologies, Inc., Santa Clara, CA, USA) maintained at 35 °C. The mobile phase was composed of acetonitrile (A, 0.1% formic acid) and water (B, 0.1% formic acid; A: B = 92:8, *v/v*) at a flow rate of 0.5 mL/min, and the injection volume was 5 L. In addition, FXOH was identified in plasma samples by using AB 5500 Q-trap LC-MS/MS (ABSCIEX, Framingham, MA, USA) equipped with electrospray ionization. Quantitative analysis of FXOH was also performed by LC-MS/MS. Detection was performed in positive ion mode under the following conditions: curtain gas at 30.0 L/h, collision gas medium, ion spray voltage at 5500 V, 550 °C, and ion source gas 1 and 2 at 40 L/h.

### 3.10. Pharmacokinetic Data Analysis

The pharmacokinetic method was consistent with that previously reported [12]. Blood drug concentration-time data were fitted with a non-compartmental model by using WinNonlin 8.1 (Certara Corporation, Princeton, NJ, USA), and the pharmacokinetic parameters of FX products were calculated and graphed. With the FX crystal suspension as the reference preparation, the relative bioavailability (Frel) of FX MCs was calculated using the formula:

(5) $$\mathrm{Frel}(\%)=\frac{{\mathrm{AUC}}_{\mathrm{FX}\;\mathrm{MC}}}{{\mathrm{AUC}}_{\mathrm{FX}\;\mathrm{crystal}}}\times 100\%$$

### 3.11. Statistical Analysis 

Graphs were constructed in OriginPro 9.0 (OriginLab Corporation, Northampton, MA, USA). Triplicate measurements were performed for each experiment. The experimental data were analyzed for significance in SPSS 9.5 (Science Press, Beijing, China). A *p* value of less than 0.05 or 0.01 was considered marginally significant or significant, respectively.

## 4. Conclusions

To enhance the stability of FX and improve its bioavailability in vivo, we utilized the nano-micro delivery system, SLN-MC, which was prepared by Ps/Chol nanoparticle wrapped with Gel/GA coacervate, using ultrasonic-assisted antisolvent precipitation. In this study, the influence of individual factors in the preparation process on the properties of microcapsules had been elucidated, in particular, the addition of Chol in SLN was found to significantly increase the LC of the microcapsules by up to 20 times. Physiochemical characterization results demonstrated that the SLN-MC carrier provides good thermal protection for FX. FX-loaded SLN-MC carrier showed better storage stability (92.01% retention of FX for 6 months). Moreover, the in vitro release showed that FX MC can be an effective means to sustain the release of FX. In addition, the relative bioavailability of FX MCs in vivo was 712.33% when compared to FX crystals, which indicated the Ps/Chol with GE/GA coating greatly improved the in vivo bioavailability of FX. In future work, a comparison study of different FX embedding preparations in vivo bioavailability will be performed. The FX-loaded SLN-MC carrier developed in this work may be an effective delivery system for application in functional foods as well as in pharmaceuticals. 

## Figures and Tables

**Table 1:** Pharmacokinetic parameters of fucoxanthinol (FXOH) after a single administration of 25 mg/kg (refers to FX content) FX crystals and FX MCs in rat plasma.

Parameters	FX Cystal	FX MC
AUC_0-∞_ [(ng/mL)·h]	856.14 ± 43.28	3871.07 ± 47.54 **
AUC_0-t_ [(ng/mL)·h]	493.89 ± 40.12	3517.89 ± 272.53 **
CL (L/h/kg)	34364.14 ± 1330.66	6515.23 ± 652.06 **
C_max_ (ng/mL)	65.42 ± 9.08	556.60 ± 46.20 **
T_1/2_ (h)	11.30 ± 2.29	7.80 ± 0.75 **
MRT_0-t_ (h)	12.87 ± 0.53	7.01 ± 0.41 **
T_max_ (h)	9.33 ± 0.81	4.67 ± 1.03 **
Vd (L)	1562721.16 ± 79156.37	304910.07 ± 24075.80 **

(*n* = 6, mean ± SD, ** *p* < 0.01 versus FX crystal suspension).

## Data Availability

All data supporting the conclusions of this article are included in this article.
